# Porcine Epidemic Diarrhea Virus among Farmed Pigs, Ukraine

**DOI:** 10.3201/eid2112.150272

**Published:** 2015-12

**Authors:** Akbar Dastjerdi, John Carr, Richard J. Ellis, Falko Steinbach, Susanna Williamson

**Affiliations:** Animal and Plant Health Agency–Weybridge, Addlestone, UK (A. Dastjerdi, R.J. Ellis, F. Steinbach);; Howells Veterinary Services Ltd, Easingwold, UK (J. Carr);; Animal and Plant Health Agency, Bury St. Edmunds, UK (S. Williamson)

**Keywords:** porcine epidemic diarrhea virus, viruses, coronavirus, Ukraine, pigs, farmed pigs

## Abstract

An outbreak of porcine epidemic diarrhea occurred in the summer of 2014 in Ukraine, severely affecting piglets <10 days of age; the mortality rate approached 100%. Full genome sequencing showed the virus to be closely related to strains reported from North America, showing a sequence identity of up to 99.8%.

Porcine epidemic diarrhea (PED), caused by PED virus (PEDV) was first recognized in the United Kingdom in 1971 (*1*). Since then, outbreaks of PED have been documented in several European and Asian countries (*2*). PED has been reported in China since the 1980s; however, in October 2010, more virulent strains of PEDV emerged there, causing high fatality rates among suckling piglets (*3*). Since May 2013, similar virulent PEDV strains have been reported in the Unites States (*4*), and a report from Ohio indicated a second incursion into the USA by a slightly different PEDV strain (USA/OH851/2014, GenBank accession no. KJ399978), characterized by focal deletions in the S gene (*5*). PEDV was subsequently reported from Mexico (*6*), Canada (*7*), and other countries in the Americas (Technical Appendix).

In May 2014, an outbreak of PED among pigs in the finishing stage of growth was reported in Germany and associated with a PEDV strain very similar to the OH851 variant of PEDV (*8*). Reports also indicate the presence of this PEDV variant in other European countries, including the Netherlands (*9*), Italy (*10*), and Spain (*11*). We investigated an outbreak of PED that occurred during the summer of 2014 in central Ukraine to determine the causative PEDV strain.

## The Study

The outbreak occurred at a large, indoor, 5,000-sow farm in the Poltava region of Ukraine where 240 sows per week were kept to give birth, which is referred to as farrowing on porcine farms. 

Clinical signs were first seen in a farrowing sow that was vomiting and had profuse diarrhea 10 days postfarrowing. Within hours, her piglets began to vomit and show profuse watery diarrhea. Vomiting and diarrhea then spread throughout the farrowing area. Disease was most severe among piglets <10 days of age; the case-fatality rate reached nearly 100%. The decision was made to euthanize piglets <10 days of age during a 3-week period from the start of the outbreak. Piglets >10 days old became sick, but most (95%) survived. Disease was less severe in adults, whose appetite returned and diarrhea ceased within 3 days. 

Abortions occurred immediately following the outbreak in 38% of 1 batch of sows that were at 28–35 days’ gestation; no diagnostic investigation was made to determine the cause. It was not established whether they were a result of the PED outbreak or control methods such as “back-feeding” fecal material to piglets. Abortions did not occur in other groups of sows and have not been reported as a feature of PED outbreaks in North America. The reproductive status of sows at other stages of gestation was not substantially affected by the initial PED outbreak, and no higher return rate was observed. Postpartum sows did not fail to produce milk, but those affected by PEDV had a reduced feed intake and associated reduced milk output. 

The performance of the pig unit took 20 weeks to return to preoutbreak levels. During that time, a total of 30,000 piglets died, which equates to a loss of 6 weaned piglets per sow per year.

The outbreak was controlled by a combination of 2 methods. Lactogenic immunity enhancement was initiated by deliberate reexposure of pregnant sows to infected piglet feces 6 weeks before farrowing. The environmental viral load was reduced by cleaning and disinfection of the area, euthanizing of neonatal piglets, and reduction of transmission by humans and other vectors through enhanced internal biosecurity. 

A diagnosis of PED was made after clinical and postmortem examination of affected piglets. We tested them onsite using a lateral flow device, Antigen Rapid PED/TGE Ag Test Kit (Bionote, Hwasung-si, Korea), which indicated the presence of PEDV antigen in the feces. We confirmed these findings at the UK Animal and Plant Health Agency using an in-house PEDV PCR and a commercially available PEDV/transmissible gastroenteritis virus quantitative reverse transcription-PCR kit (QIAGEN, Hilden, Germany). A BLAST search (http://blast.ncbi.nlm.nih.gov/Blast.cgi) of the 160-nt PCR amplicon revealed the highest similarity (99%) to PEDV strains from the United States and China. PEDV RNA was then subjected to deoxyribonuclease I digestion and converted to cDNA for preparation of sequencing libraries by using a Nextera XT kit (Illumina, San Diego, CA, USA). Paired-end sequencing was performed on an Illumina MiSeq. The consensus sequence was obtained by de novo assembly by using the Velvet 1.2.10 algorithm (*12*) of the sequence reads that mapped to the reference strain (GenBank accession no. NC003426).

The PEDV Ukraine/Poltava01/2014 strain genome (GenBank accession no. KP403954) is 27823 nt (excluding the 3′ poly A tail). Nucleotide analyses of the full genome of the virus showed the highest similarity to PEDV strains reported in 2013 from the United States; specifically, strains USA/Kansas29/2013 (GenBank accession no. KJ645637.1) and USA/Colorado30/2013 (GenBank accession no. KJ645638.1), with 99.8% nucleotide identity. The nucleotide identity was substantially lower (98.5%) when compared to those of the 2014 strains isolated in Germany, which are similar to another strain isolated in North America, Ohio851/2013 (*8*). Accordingly, the Ukraine virus clustered phylogenetically with PEDV strains from North America in genetic clade II (*6*) but was distinct from strains currently and previously found elsewhere in Europe, such as the prototype PEDV strain CV777, with which it shares only 96.5% homology (Figure [*13*]). However, the genetic analysis does not, at this stage, support drawing conclusions regarding the relative pathogenicity of this apparently virulent PEDV strain, similar to past instances of PEDV in Europe, of which very few have been characterized; parallel experimental infection of pigs would be required for further investigation.

**Figure:**
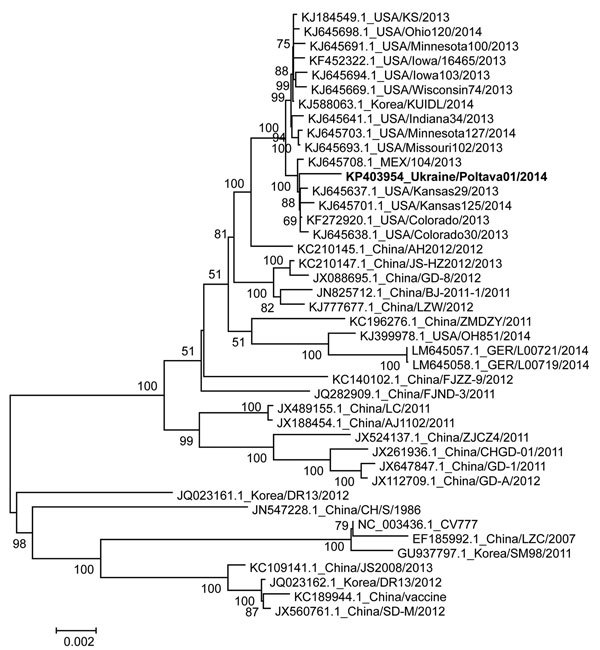
Phylogenetic analysis of the full-length genome of the porcine epidemic diarrhea virus (PEDV) Ukraine/Poltava01/2014 (bold text). The full-length genomes of PEDV were aligned by using the MegAlign software of the DNASTAR Lasergene Core Suite (DNASTAR, Inc., Madison, WI, USA) and phylogenetic analysis was done by using MEGA 5.2 software (*13*). The tree was constructed by using the neighbor-joining method and 1,000 bootstrap replications. Only bootstrap values of more than 50% are shown in the figure. Each virus on the tree is represented by accession number, strain, and year of sample collection. Ukraine/Poltava01/2014 clusters in close proximity to recent strains in the United States other than the Ohio851 variant, and both are substantially genetically different from the previous European variants, such as the prototype strain CV777, which is embedded in another cluster. Scale bar indicates nucleotide substitutions per site.

The farm was found to be free from *Mycoplasma hyopneumoniae*, *Brachyspira hyodysenteriae*, mange, toxigenic *Pasteurella multocida,* porcine reproductive and respiratory syndrome virus, transmissible gastroenteritis virus, Aujeszky’s Disease virus, and classical and African swine fever viruses. Persons on the farm practiced strict biosecurity measures to maintain a specific pathogen free status. No breach in biosecurity could be identified after a review of potential PEDV introduction routes involving pigs or humans or vehicles, equipment, and other fomites; the source of infection remains unknown. However, there were reports of a PED-affected pig farm 1.5 km from this unit, and the potential for unidentified fomite or other transmission from this herd, or another undisclosed infected herd, therefore existed. Windborne transmission has been suggested to explain some outbreaks in the United States (*14*) and is another possibility to consider in this case.

## Conclusions

This PED outbreak in Ukraine showed clinical characteristics similar to outbreaks caused by virulent strains of PEDV reported from North America (*4*). The virus clusters phylogenetically with viruses from recent outbreaks in the United States and Mexico (*6*). The presence of such a PEDV strain in Ukraine highlights the threat to neighboring countries and those in the European Union where PEDV has not been detected (e.g., Scandinavia) or has not caused disease in recent decades (e.g., the United Kingdom) and where pig herds are considered largely naive to PEDV. Furthermore, pig farming has changed over recent decades, and the establishment of more large holdings would produce more virus following introduction of PEDV. 

This outbreak emphasizes the need for decision-makers of countries, pig farms, and allied industries to implement and maintain biosecurity measures to minimize the risk for spread of PEDV to new areas. Early detection of suspected clinical signs on pig farms and, when these occur, prompt testing for PEDV by PCR are also vital (*15*). A coordinated approach is essential to prevent introduction of PEDV, promote early detection should introduction occur, control disease, and minimize spread of infection.

Technical Appendix References for outbreaks of porcine epidemic diarrhea virus in 3 countries in the Americas during 2014.
