# Supplementary material for: Porcine Epidemic Diarrhea Virus among Farmed Pigs, Ukraine
Source: Emerg Infect Dis. 2015 Dec;21(12):2235–7. doi: 10.3201/eid2112.150272 (PMC4672447; doi:10.3201/eid2112.150272)
Supplement: Technical Appendix — References for outbreaks of porcine epidemic diarrhea virus in 3 countries in the Americas during 2014. [file 15-0272-Techapp-s1.pdf]

Article DOI: http://dx.doi.org/10.3201/eid2112.150272

Porcine Epidemic Diarrhea Virus among Farmed Pigs, Ukraine

Technical Appendix

Porcine Epidemic Diarrhea Outbreaks in the Americas, 2014

Porcine epidemic diarrhea virus outbreaks were reported in Columbia, the Dominican Republic, and Ecuador during 2014. See the following ProMED articles (http://www.promedmail.org/): Columbia, archive nos. 20140319.2341300 and 20140611.2532426; Dominican Republic, archive no. 20140616.2544899; Ecuador, archive no. 20140910.2763771.
